# Detrusor Induction of miR-132/212 following Bladder Outlet Obstruction: Association with MeCP2 Repression and Cell Viability

**DOI:** 10.1371/journal.pone.0116784

**Published:** 2015-01-24

**Authors:** Mardjaneh Karbalaei Sadegh, Mari Ekman, Katarzyna Krawczyk, Daniel Svensson, Olga Göransson, Diana Dahan, Bengt-Olof Nilsson, Sebastian Albinsson, Bengt Uvelius, Karl Swärd

**Affiliations:** 1 Department of Experimental Medical Science, Lund University, Lund, Sweden; 2 Department of Clinical Sciences, Lund University, Lund, Sweden; 3 Department of Biology, Lund University, Lund, Sweden

## Abstract

The microRNAs (miRNAs) miR-132 and miR-212 have been found to regulate synaptic plasticity and cholinergic signaling and recent work has demonstrated roles outside of the CNS, including in smooth muscle. Here, we examined if miR-132 and miR-212 are induced in the urinary bladder following outlet obstruction and whether this correlates with effects on gene expression and cell growth. Three to seven-fold induction of miR-132/212 was found at 10 days of obstruction and this was selective for the detrusor layer. We cross-referenced putative binding sites in the miR-132/212 promoter with transcription factors that were predicted to be active in the obstruction model. This suggested involvement of Creb and Ahr in miR-132/212 induction. Creb phosphorylation (S-133) was not increased, but the number of Ahr positive nuclei increased. Moreover, we found that serum stimulation and protein kinase C activation induced miR-132/212 in human detrusor cells. To identify miR-132/212 targets, we correlated the mRNA levels of validated targets with the miRNA levels. Significant correlations between miR-132/212 and *MeCP2, Ep300, Pnkd* and *Jarid1a* were observed, and the protein levels of MeCP2, Pnkd and Ache were reduced after obstruction. Reduction of Ache however closely matched a 90% reduction of synapse density arguing that its repression was unrelated to miR-132/212 induction. Importantly, transfection of antimirs and mimics in cultured detrusor cells increased and decreased, respectively, the number of cells and led to changes in MeCP2 expression. In all, these findings show that obstruction of the urethra increases miR-132 and miR-212 in the detrusor and suggests that this influences gene expression and limits cell growth.

## Introduction

MicroRNAs (miRNAs) are evolutionarily conserved small RNA molecules (19–24 nucleotides) that hybridize with mRNAs leading to degradation or translational repression [1]. The ribonuclease activities of Dicer and Drosha are required for processing of miRNA precursors [2]. In recent work, using conditional and smooth muscle-specific deletion of Dicer, we established roles of miRNAs in contractility, matrix deposition and cholinergic neuro-effector transmission in the urinary bladder [3]. In subsequent work we discovered that miR-29b and miR-29c key roles in extracellular matrix homeostasis, and, using microarrays, we found over 50 miRNAs that were differentially expressed following outlet obstruction [4]. These findings support the notion that miRNAs play important roles in urological pathologies beyond cancer.

In humans, miR-132 and miR-212 are derived from a bicistronic precursor transcribed from chromosome 17 [5]. MiR-132 and miR-212 have identical seed sequences and are therefore predicted to target an overlapping set of mRNAs. MiR-132 and miR-212 are expressed in neurons where they regulate excitability [6], and a body of literature has demonstrated roles of these miRNAs in synaptic plasticity [7], [8], [9]. One target of miR-132/212 is MeCP2 [10], a methyl CpG-binding protein that controls transcription and that is mutated in the developmental disorder Rett syndrome [11]. Recent work has highlighted roles of miR-132 and miR-212 in non-neural tissues. For example, induction of miR-132 in lipopolysaccharide-challenged splenocytes was shown to repress acetylcholine esterase (*Ache*) leading to cholinergic suppression of inflammation [12]. It has also been demonstrated that miR-132 promotes proliferation and tube-formation in endothelial cells by targeting p120RasGAP (*Rasa1*, [13]). Work in the heart, finally, has shown that miR-132 and miR-212 are involved in hypertrophy [14]. These findings point to pleiotropic roles of these miRNAs in multiple cell types.

Early work established that the transcription factors REST and CREB are involved in the transcriptional control of the miR-132/212 cluster [6], [15], [16]. Intriguingly, expression of miR-132/212 is also regulated by DNA methylation and MeCP2 [17]. Because MeCP2 has a dual role as a regulator and a target of these miRNAs [10], a feed-forward loop may exist that forms the basis for rapid and surge-like changes in the expression of this miRNA cluster. Recent experiments have established an impact of the dioxin receptor (*Ahr*) on miR-132/212 expression in T cells [18]. Histone modifications have also been shown to play a role [9]. Despite these advances, the transcriptional control of the miR-132/212 cluster probably remains incompletely elucidated [5].

Recent studies have indicated roles of miR-132/212 in smooth muscle. It was shown that angiotensin II upregulates miR-132/212 in arterial smooth muscle leading to reduction of phosphatase and tensin homolog (PTEN) [19]. These findings are in accord with results in human hypertension [20]. MiR-132 was also upregulated by arterial balloon injury [21], and this was replicated by PDGF in cell culture. These latter studies showed that transfection of miR-132 mimic led to reduced smooth muscle cell viability, proliferation and migration [21], suggesting the existence of a negative feedback loop in which growth-promoting stimuli increase miR-132 and miR-212 which in turn limit cell proliferation.

When surveying miRNA arrays from the obstructed urinary bladder [4] we noted changes in miR-132/212. Here, we aimed to document and confirm these changes and to explore possible downstream consequences of miR-132/212 induction. In view of the cholinergic neuro-effector transmission defect that we have reported for the bladder following smooth muscle-specific deletion of Dicer [3], we also tested if miR-132/212 regulates cholinergic activation of the bladder via effects on acetylcholine esterase (*Ache*). Our work suggests that outlet obstruction increases miR-132/212 via the transcription factors Ahr and Creb and that these miRNAs repress MecCP2 and limit cell growth. To the best of our knowledge, our studies are the first to document induction of miR-132/212 and attendant changes in target mRNA levels in the detrusor following outlet obstruction.

## Materials and Methods

### Ethics statement

The procedures were approved by Malmö-Lunds Djurförsöksetiska Nämnd (regional animal ethics committee) and Regionala Etikprövningsnämnden (regional human ethics committee) and the ethic permit numbers are M300-08, M212-13, M259-11 and 2008-4 (human). Procedures adhere to principles expressed in the Declaration of Helsinki. Written informed consent was obtained from patients donating bladder tissue at cystectomy. Ketamine and xylazin were used for animal anesthesia as described [4] and animals were euthanized using increasing CO_2_.

### Surgery

The urethra of female Sprague-Dawley rats (≈12 weeks old weighing 200g, Taconic Europe, Denmark) was partially obstructed as described [4]. The experimental series used for the microarrays ([4]; GEO accession GSE47080) consisted of rats obstructed for 10 days and for 6 weeks. Another group of rats were first obstructed and then re-operated (at 6 weeks) to remove the obstruction. Those animals were sacrificed 10 days later. In the present work we included rats that had been obstructed for 2, 4 and 10 days and for 6 weeks, as well as sham-operated controls. Following sacrifice, bladders were removed, weighed and frozen in liquid nitrogen. In one series, the detrusor and mucosa were separated by micro-dissection in ice cold Ca^2+^-free HEPES-buffered Krebs solution (sodium chloride, 135.5 mM; potassium chloride, 5.9 mM; magnesium chloride, 1.2 mM; glucose 11.6 mM; HEPES, 11.6 mM; pH 7.4 at 37°C) under a dissection microscope. The detrusor and mucosal layers were then frozen separately.

### Dicer KO mice

Mice with smooth muscle-specific inactivation of Dicer were bred at the local animal facility. At the age of 4 weeks they were given intraperitoneal injections of tamoxifen or vehicle (1:10 EtOH in sunflower oil) as described in the original report on these mice [22]. Following euthanization at 14 weeks, the bladder was removed and the detrusor and mucosa were separated by micro-dissection. Tissue was then frozen for isolation of RNA and protein. Alternatively, detrusor strips were prepared for electrical field stimulation as outlined below.

### Quantitative real-time PCR

RNA was isolated using miRNeasy mini kit (Qiagen, Valencia, CA #217004) and 300 ng of RNA was reverse transcribed to cDNA using miScript II RT Kit (Qiagen, #218161). Expression of miRNAs was measured by a real time thermal cycler (StepOnePlus, Applied Biosystems) using miScript SYBR Green PCR Kit (Qiagen, # 218073) and miScript Primer Assays (for miR-132, miR-212, miR-143, miR-145, miR-1, SNORD-68, SNORD-72, U6, 18S) as described [4]. *Ache* mRNA expression was determined using Quantitect (Qiagen) primer assays [4] and *Gapdh* was used as housekeeping gene.

### Transcription factor binding site analysis

Transcription factor binding site (TFBS) analysis exploits gene lists from microarray experiments to predict involvement of transcription factors in the observed expression changes. The analysis returns a probability to acquire the observed number of binding motifs in differentially expressed gene promoters by a resampling and ranking procedure. TFBS analysis was used as described [4] to identify transcription factors whose binding motifs were significantly enriched at 10 days of outlet obstruction. p<0.05 was considered significant.

### Western blotting

Sham-operated and obstructed bladders (2 days, 4 days, 10 days and 6 weeks) as well as bladders from vehicle- and tamoxifen-treated Dicer KO mice were used. Homogenization, protein determination, gel electrophoresis and transfer to nitrocellulose membranes were performed as described [3,23]. 25 μg protein was loaded per lane. Membranes were cut in horizontal strips and incubated with the indicated primary antibodies. Some membranes were stripped using Restore PLUS Western blot stripping buffer (Thermo Scientific). Table 1 lists the primary antibodies used. Dilutions were optimized in each case. Arrowheads in the figs indicate the expected molecular weight of the antigen. Secondary antibodies were horseradish peroxidase-conjugated (1:5000 to 1:20 000 dilutions; 7074, 7076; Cell signaling; ab97120; Abcam) and we used West Femto reagent (Pierce, Rockford, IL, USA) for chemiluminescence detection. Images were acquired using the LI-COR Odyssey Fc equipment (LI-COR Biosciences, Lincoln, NE, USA). The signal of the band of interest was normalized to that of β-actin in the same lane. These values were then normalized to the mean of the sham operated controls.

**Table 1:** Primary antibodies used in the study are listed in the order that they appear in the figures.

**Name**	**Product number**	**Host**	**Company**
**Ahr**	MA1-514	Mouse	Thermo Scientific
**Ahr**	Sc-8088	Goat	Santa Cruz
**β-actin**	A5441	Mouse	Sigma
**P-CREB (S133)**	#9198	Rabbit	Cell Signaling
**CREB**	#9197	Rabbit	Cell Signaling
**Ache**	Sc-6431	Goat	Santa Cruz
**MeCP2**	#3456	Rabbit	Cell Signaling
**Pten**	#9552	Rabbit	Cell Signaling
**Ripk2**	Ab108971	Rabbit	Abcam
**Sh3bp5**	H00009467-M01	Mouse	Novus Biologicals
**Pnkd**	HPA010134	Rabbit	Atlas antibodies
**Cav1**	#3267	Rabbit	Cell Signaling
**P-Akt**	#9271	Rabbit	Cell Signaling
**T-Akt**	#9272	Rabbit	Cell Signaling
**P-ERK**	#9101	Rabbit	Cell Signaling
**T-ERK**	#9102	Rabbit	Cell Signaling
**phospho-PKA substrate**	#9621	Rabbit	Cell Signaling

### Cell culture, stimulation and transfection

Smooth muscle cells were isolated from cystectomized human bladders as described [4],[24]. Cells in passages 2–6 were cultured in DMEM/Ham’s F12 supplemented with antibiotics (penicillin and streptomycin) and 10% fetal calf serum (FCS). The cells were treated for 24h with the following agents: 2,3,7,8-tetrachlorodibenzo-p-dioxin (TCDD, 0.16 μM 48599, Sigma Aldrich), forskolin (10 μM, F6886, Sigma Aldrich), brefeldin A (5 μg/ml, B7651 Sigma Aldrich), tunicamycin (5 μg/ml, T7765, Sigma Aldrich), Phorbol 12-myristate 13-acetate (PMA, 1μM, P8139, Sigma Aldrich) or fetal calf serum (FCS). Cells were harvested and RNA isolated as described above. MiR-132 and miR-212 inhibitors (AM10166, AM10340, Ambion, Thermo Scientific, Pittsburgh, PA, USA; 10 nM and 100 nM), mimics (Mission miRNA: Sigma-Aldrich, St. Louis, MO, USA; 10 and 100 nM) and negative control (HMC0002, Mission miRNA: Sigma-Aldrich, St. Louis, MO, USA; 10 and 100 nM) were transfected using Oligofectamine reagent (Life Technologies). Successful transfection of miR-132/212 mimics and inhibitors was confirmed using real-time quantitative PCR. Experiments with human cells are from 3–7 independent replicates using cells from both males and females.

### Cell viability and proliferation

Cells were plated at a density of 4.0×10^3^ cells/well of 96-well plates. After 24 h of culture, cells were transfected. Medium was subsequently replaced with 100 μl of serum-free DMEM and 10 μl of 3-(4,5-dimethylthiazol-2-yl)-2,5-diphenyl tetrazolium bromide (MTT) solution (5 mg/ml MTT in PBS), after which the 96 well plates were returned to the incubator for 1 h. The MTT containing medium was removed and the blue formazan product was dissolved by adding 100 μl of DMSO per well. Absorbance was monitored at 540 nm in a Multiscan GO Microplate Spectrophotometer (Thermo Scientific). Cell count and viability were measured using the Luna Automated Cell Counter system (Logos Biosystem, USA) in accordance with the manufacturer’s instructions.

### Electrical field stimulation

Bladders from control and Dicer KO mice was excised and dissected in cold Ca^2+^-free HEPES buffered Krebs. The urothelium was removed and two smooth muscle strips from the middle section of the urinary bladder was prepared and mounted in open organ bath filled with Krebs solution (in mM: NaCl 119, KCl 4.6, NaH_2_PO_4_ 1.2, NaHCO_3_ 15, MgCl_2_ 1.2, glucose 5.5 and CaCl_2_ 1.5) and connected to a force transducer (Grass FT03C; Grass Medical Instruments). To maintain pH at 7.4, the organ baths were gassed with 95% O_2_ and 5% CO_2_ at 37°C. Bladder strips was allowed to equilibrate for 30 min and then stretched to obtain L_0_ (optimum length of force development). Each experiment was started and ended by depolarization using 60 mM K^+^ HEPES buffered Krebs. Electrical field stimulation was performed by placing electrodes at each side of smooth muscle tissue and stimulating the intramural nerves in the bladder with at increasing frequency (1–50 Hz) at the optimum voltage for force development. The experiment was repeated in the presence of 10μM α,β-methylene-ATP and 3μM neostigmine (M6517, N2001 Sigma Aldrich) applied 30 min prior to stimulation.

### Immunofluorescence

Bladders were fixed, sectioned and mounted [23] and 10-μm thick transmural sections were incubated overnight (4°C) with rabbit MeCP2 (1:500, Table 1) or mouse Ahr antibodies (1:50) in phosphate-buffered saline (PBS; pH 7.2) containing 0.25% bovine albumin and 0.25% Triton X-100. After rinsing and incubation with anti-rabbit secondary antibody coupled to Alexa fluor 488 nitrogen (1:500, Molecular Probes, Eugene, OR, USA) nuclei were stained with bisbenzimide (1 μg/ml). Micrographs were acquired at identical settings in a fluorescence microscope (Olympus AX70TRF; Olympus, Tokyo, Japan). ImageJ was used to define and count MeCP2 positive nuclei in micrographs from 6 sham-operated and 7 obstructed rats (10 days).

### Electron microscopy

Six control and six obstructed bladders (6 weeks) were excised and transferred to fixative (2.5% glutaraldehyde in 150 mM sodium cacodylate, pH 7.4) without emptying. After 30 min they were opened longitudinally and transferred to new fixative. After 2 h the mid-ventral part of the bladders was dissected and transferred to fresh buffer. Longitudinal strips were cut, and post-fixed in 1% osmium tetroxide for 2h, block-stained with uranyl acetate, and embedded in Araldite. Sections were then cut and examined in a microscope. Cutting planes that were ortogonal to the long axis of the smooth muscle cells were then chosen. Thin sections were cut for electron microscopy using a JEOL JEM 1230 microscope (Jeol, Tokyo, Japan). The cross-sectional area of the muscle bundles was measured on micrographs acquired at 5K magnification using ImageJ (NIH, Bethesda, MD, USA), and the number of nerve terminals within the bundles was counted. The muscle bundle area measured for each bladder was between 8016 and 14122 μm^2^. The nerve terminal density is expressed as number of terminals per 100 μm^2^ muscle bundle cross sectional area.

### Statistical analysis

GraphPad Prism version 5.02 for Windows (GraphPad Software, San Diego California USA) was used for statistical analyses. Pair-wise comparisons were made using Student’s t test. Multiple comparisons were made with analysis of variance followed by Bonferroni’s or Tukey’s multiple comparison tests. For miRNA-mRNA correlations, fold expression changes (vs. sham) were retrieved from our recent microarray experiments (GEO accession GSE47080) and correlations were tested using linear regression analysis. A *p*-value <0.05 was considered significant.

## Results

### Induction of miR-132 and miR-212 following bladder outlet obstruction

**Figure 1:**
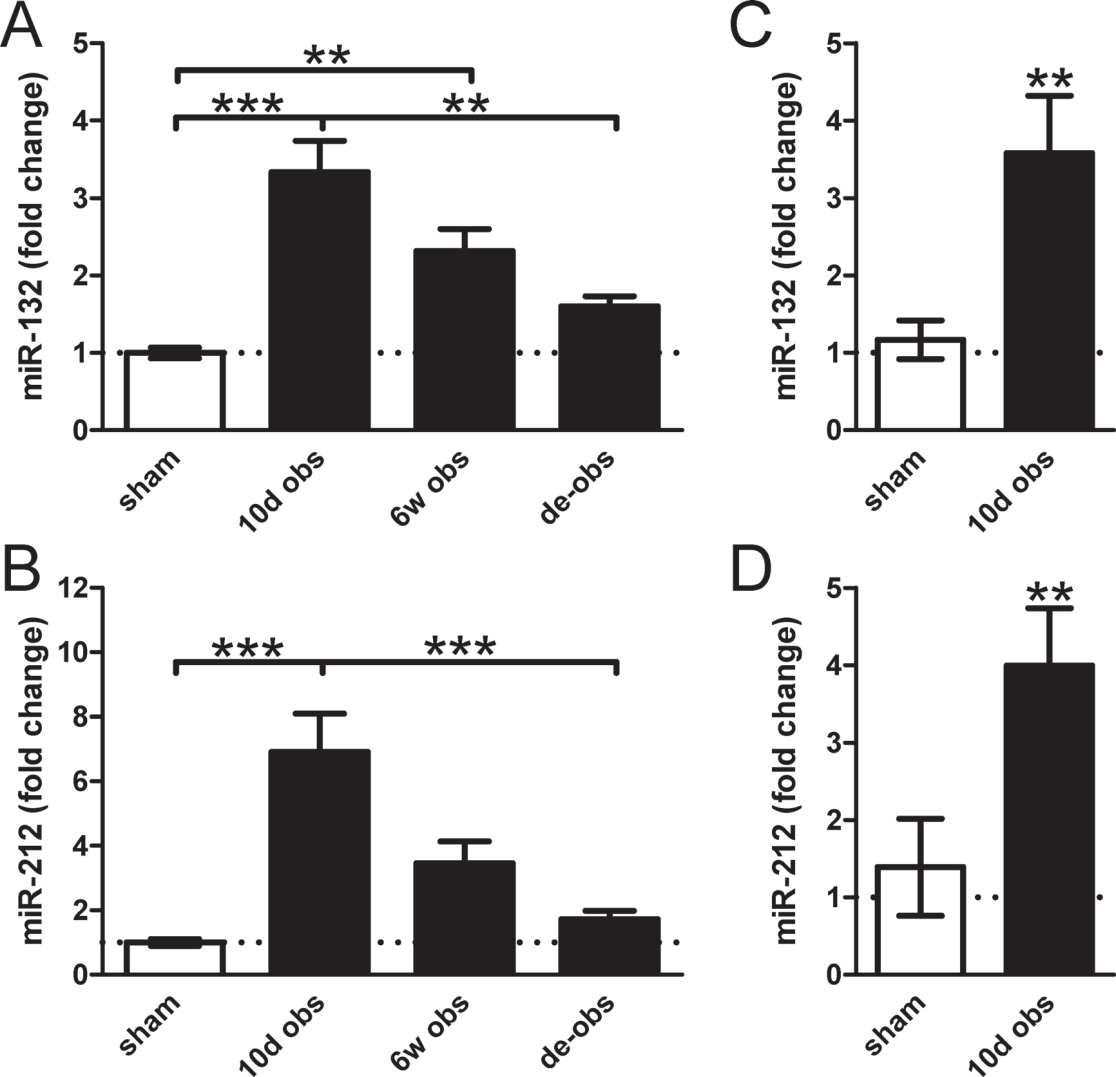
Partial infravesical outlet obstruction increases expression of miR-132/212 in the urinary bladder. Rats were sham-operated (sham) or subjected to obstruction for 10 days (10d obs) and 6 weeks (6w obs). One group of rats were obstructed for 6 weeks and then re-operated to remove the obstruction. The obstruction-induced hypertrophy was then allowed to regress for another 10 days (de-obs). Bladders were harvested, RNA was isolated and microarrays were run (GEO accession GSE47080). Results in panels **A** and **B** are from these microarrays. Panels **C** and **D** show confirmation using qRT-PCR at 10 days of obstruction versus sham. *, **, and *** denote p<0.05, p<0.01 and p<0.001 in this and the following figures. N = 6–8 throughout.

Microarrays using bladders obstructed for 10 days and at 6 weeks as well as de-obstructed and sham-operated bladders (GEO: GSE47080) demonstrated induction of miR-132 (rno-miR-132-3p) and of miR-212 (rno-miR-212-3p) in bladder outlet obstruction and a return towards control level on de-obstruction (Fig. 1A, B). The corresponding star sequences (5p) were similarly induced (rno-miR-132-5p: 3.2-fold, *p* = 0.0005; rno-miR-212-5p: 4.0-fold, *p* = 0.0001). The changes at 10 days were confirmed using qRT-PCR (Fig. 1C, D).

### MiR-132 and miR-212 are increased in the detrusor and not in the mucosa following outlet obstruction

**Figure 2:**
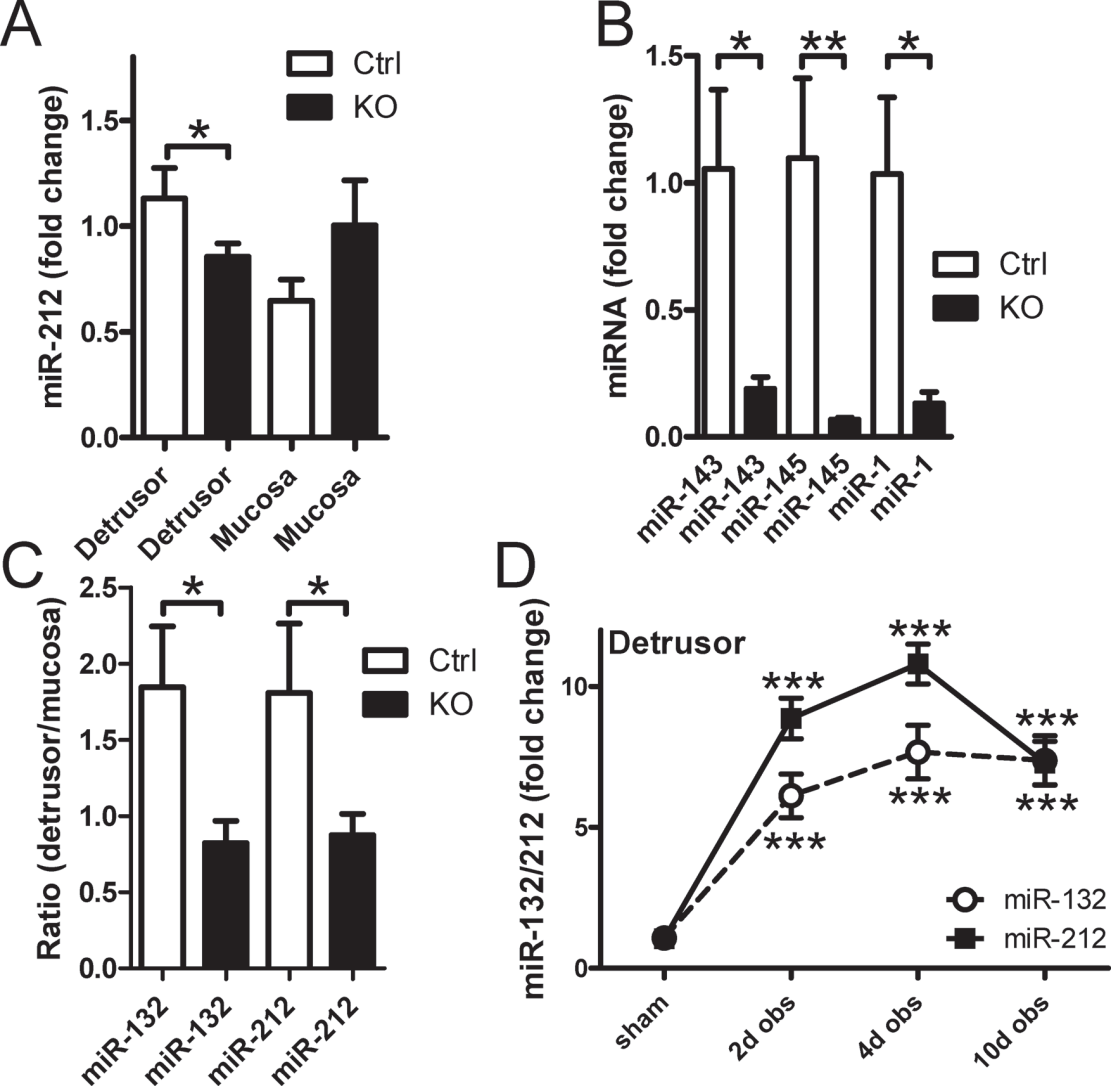
Basal expression of miR-132/212 in detrusor smooth muscle is low yet induction following outlet obstruction occurs only in the detrusor layer. Control mice and mice with smooth muscle-specific deletion of Dicer (KO) were used to examine the contribution of smooth muscle cells to miR-132 and miR-212 expression in the bladder (panels **A** through **C**). Panel **A** shows miR-212 expression in control and KO mice following separation of the detrusor and mucosa using micro-dissection. Panel **B** shows expression of miR-143, miR-145 and miR-1 in control and KO detrusor. Panel **C** shows the detrusor to mucosa expression ratio of miR-132 and miR-212 in control and KO bladders. Time-courses of miR-132/212 expression in detrusor following partial bladder outlet obstruction in the rat are shown in panel **D**. N = 4–10 throughout.

In order to examine in which tissue compartment miR-132 and miR-212 are expressed, we used control and smooth muscle-specific Dicer knockout (KO) mice. The mucosa and detrusor layer were separated by micro-dissection followed by RNA isolation and qRT-PCR analysis. Despite the fact that miR-132 and miR-212 are Dicer-dependent miRNAs, only small (≈24%) reductions in miR-212 (*p* = 0.03, Fig. 2A) and miR-132 (*p* = 0.08, not shown) were observed in Dicer KO detrusor. This contrasted with miR-143, miR-145 and miR-1 which were all reduced by at least 75% in the detrusor from KO mice (Fig. 2B). We reasoned that variable mRNA breakdown during dissection could play a role for the lack of a small effect of Dicer deletion on miR-132. This should be cancelled by using the detrusor:mucosa expression ratio because similar degradation is expected in the two tissue layers from the same bladders as dissection time, trauma etc. are identical. We found that the detrusor:mucosa ratios of both miR-132 and miR-212 were reduced in Dicer KO bladders (Fig. 2C). Together, these findings argue that miR-132/212 in the control bladder is expressed primarily by cells other than smooth muscle cells, or, alternatively, that these miRNAs are comparatively stable.

Using the same micro-dissection approach we next determined if miR-132/212 induction occurred in the detrusor or in the mucosa in obstructed rat bladders. The outcome of this experiment was clear-cut, with a 7–10-fold induction in the detrusor (Fig. 2D) and no significant change in the mucosa (not shown). This demonstrates that miR-132/212 induction in outlet obstruction is specific for the detrusor layer.

### A role for Ahr in miR-132/212 induction

**Figure 3:**
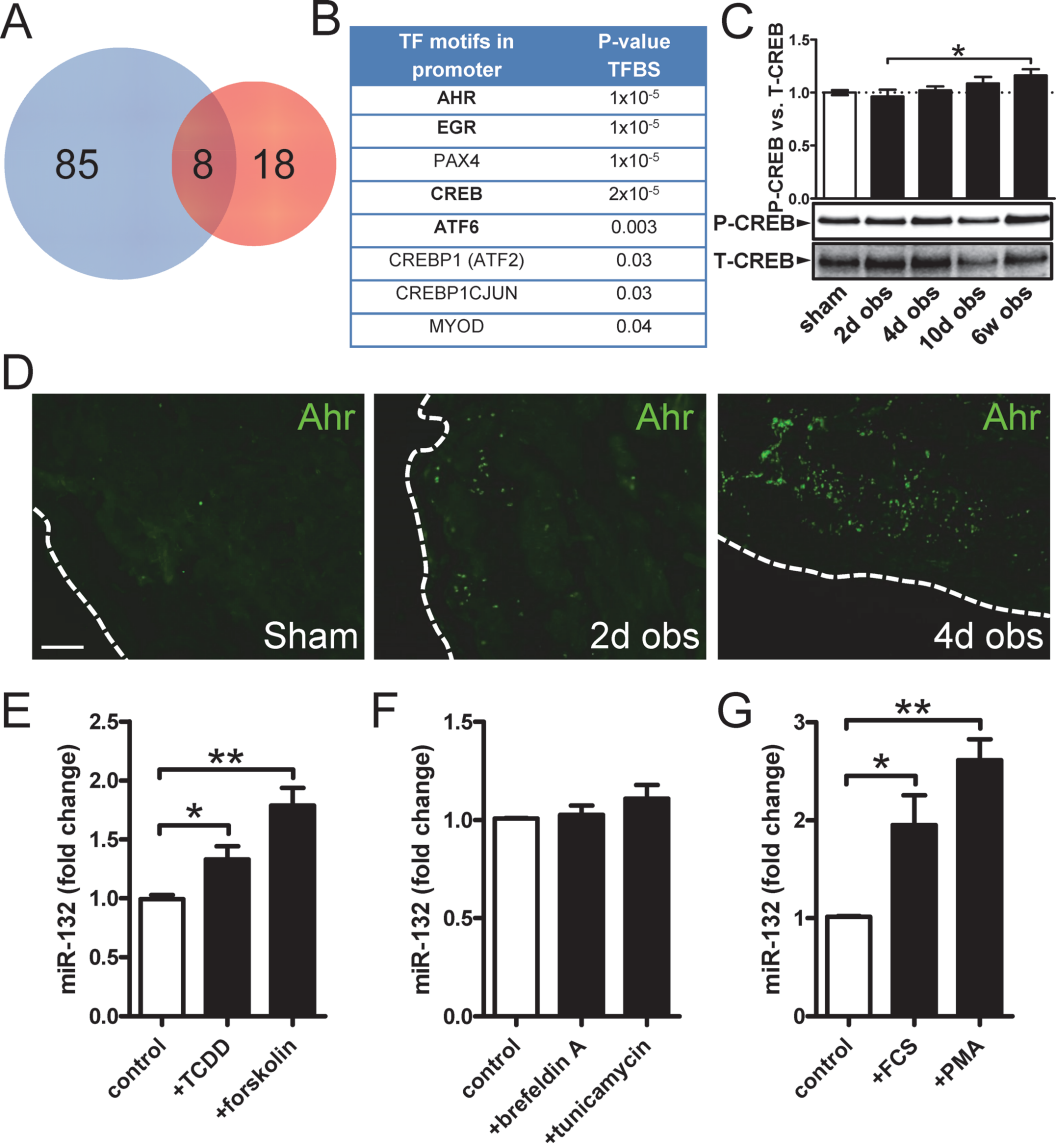
Bioinformatics analysis points to the involvement of Ahr in miR-132/212 induction following outlet obstruction. Transcription factor binding site (TFBS) analysis identified significant enrichment of 93 transcription factor binding motifs in promoters of differentially expressed genes at 10 days of obstruction (blue circle in **A**). These were cross-referenced against putative transcription factor binding sites in the miR-132/212 promoter (red circle in **A**), yielding a list of putative mediators of miR-132/212 induction in outlet obstruction (**B**). Hits with low expression levels and those with raw p-values exceeding 0.01 are shown in thin lettering. Panel **C** shows phosphorylation of CREB (S133) in bladders from sham-operated rats and following various time of obstruction. Panel **D** shows immunofluorescence labeling (green) of the dioxin receptor Ahr in bladders from sham-operated and obstructed rats. Dotted lines indicate the outer surface of the urinary bladder. The scale bar to the left applies to all images and represents 100 μm. Panels **E** through **G** show miR-132 expression in human bladder smooth muscle cells stimulated with vehicle (control) and various pharmacological substances in vitro. TCDD: 2,3,7,8-tetrachlorodibenzo-p-dioxin; FCS: fetal calf serum; PMA: Phorbol 12-myristate 13-acetate. Further details are given in Materials and Methods.

Transcription factor binding site (TFBS) analysis is an indirect method to predict involvement of transcription factors using gene lists from microarray experiments. TFBS analysis at 10 days of obstruction (vs. sham) supported involvement of 93 (85+8, Fig. 3A, blue circle) transcription factors in the expression changes resulting from obstruction. We cross-referenced these against putative binding sites in the miR-132/212 promoter (Fig. 3A red circle). Eight transcription factors were represented in both circles of the Venn diagram (Fig. 3A). This list (Fig. 3B) was further shortened by removing transcription factors with expression levels equaling the negative controls in sham-operated and obstructed bladders. The top remaining candidates were the dioxin receptor (*Ahr*), Early growth response 1 and 2 (*Egr1/2*), CREB (*Creb1*) and ATF6α (*Atf6*).

Prior evidence, in other cell types, for involvement of *Creb1* and *Ahr* in miR-132/212 expression exists so we initially focused on these. Creb1 phosphorylation was not increased at 4 days when miR-132/212 peaked (Fig. 3C, compare with Fig. 2D). This was not due to dephosphorylation following freezing because AKT, ERK1/2 and PKA substrate phosphorylation were increased at 2 days (data not shown). Whereas Ahr staining was cytoplasmic in sham-operated bladders, a clear-cut increase in the number of Ahr positive nuclei was seen at 2 and, especially, at 4 days of outlet obstruction (green in Fig. 3D). Western blotting did not support increased Ahr protein expression at either 2 or 4 days (not shown) arguing that increased nuclear fluorescence intensity after obstruction reflects shuttling of Ahr from the cytoplasm to nuclei. We next stimulated human bladder smooth muscle cells with the Ahr agonist TCDD. This resulted in a modest but significant induction of miR-132 (Fig. 3E). This supported involvement of Ahr alone or in combination with some other factor. Forskolin, which activates Creb, induced miR-132 effectively in bladder smooth muscle cells (Fig. 3E). ATF6 is activated by endoplasmic reticulum (ER) stress, but we did not see induction of miR-132 following stimulation with the ER stressors brefeldin A or tunicamycin (Fig. 3F). Serum stimulation (FCS) and phorbol ester (PMA) on the other hand increased miR-132 expression (Fig. 3G). Largely similar results for all drugs used in Fig. 3E through G were obtained for miR-212 with one exception; brefeldin A significantly suppressed miR-212 (*p* = 0.045, not shown). In summary, this analysis suggested involvement of Ahr in miR-132/212 induction in the obstructed bladder.

### Correlations between miR-132/212 and target mRNAs

**Figure 4:**
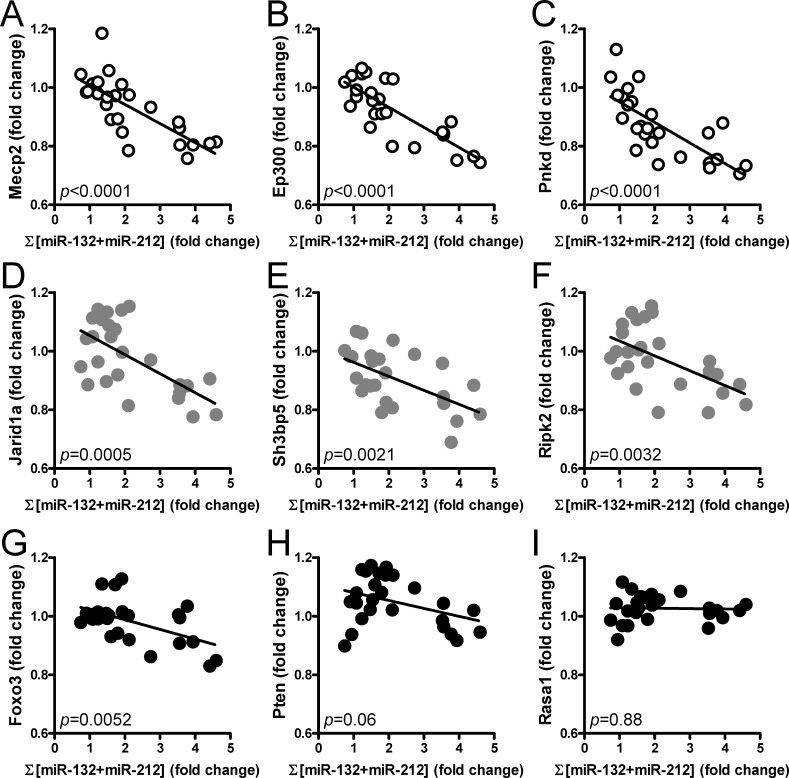
MiR-132/212 induction in the bladder correlates inversely with previously validated miR-132/212 targets. The normalized sum of miR-132 and miR-212 in bladders from sham-operated, obstructed (10 days and 6 weeks) and de-obstructed rats was correlated with the mRNA levels for *Mecp2* (**A**), *Ep300* (**B**), *Pnkd* (**C**), *Jarid1a* (**D**), *Sh3bp5* (**E**), *Ripk2* (**F**), *Foxo3* (**G**), *Pten* (**H**) and *Rasa1* (**I**). Expression data were obtained from microarray experiments (GEO accession GSE47080). Each symbol represents one rat and the 10d obstructed rats are represented by the rightmost symbols. The *p*-values obtained for the individual correlations are given at the bottom left in each diagram.

We next searched the literature for validated miR-132/212 targets and selected nine for which correlations with miR-132 and miR-212 were examined. Highly significant correlations in the expected directions were found for *Mecp2, Ep300* and *Pnkd* (Fig. 4A-C). Correlations were significant also for *Jarid1a, Sh3bp5, Ripk2* and *Foxo3* (Fig. 4D-G). *Pten* and *Rasa1* did not correlate with the miR-132/212 level (Fig. 4H-I). *Ache* was filtered due to its low expression level, and was not examined.

### MeCP2, Pnkd and Ache are reduced at the protein level in outlet obstruction

**Figure 5:**
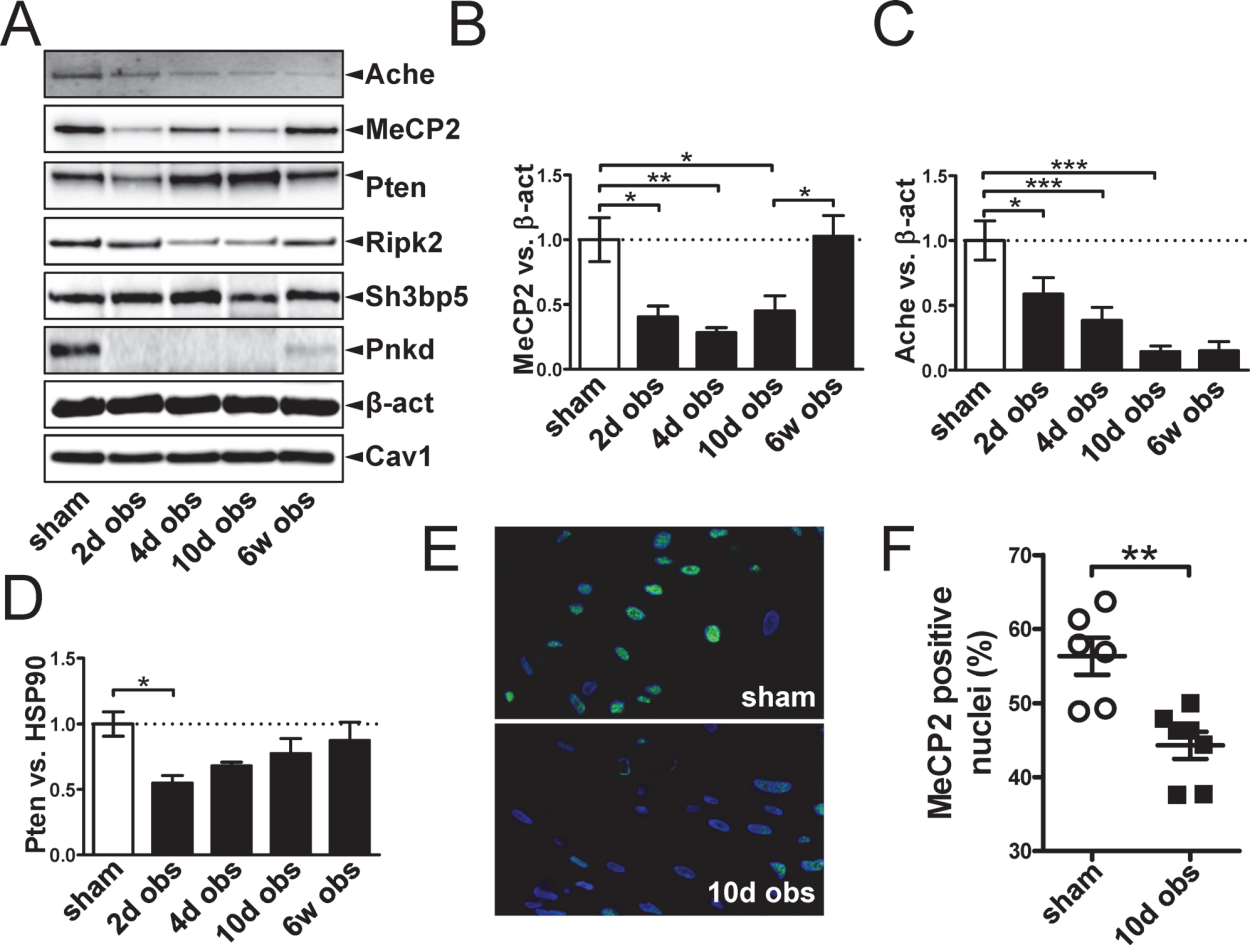
Reduced levels of the miR-132/212 targets Ache, MeCp2 and Pnkd in the bladder following outlet obstruction. Validated targets of miR-132/212 were examined at the protein level using western blotting at various times after surgical obstruction of the urethra (**A**). Summarized data (n = 6) for MeCP2, Ache and Pten is shown in panels **B** through **D**. Panel **E** shows double staining for MeCP2 (green) and DNA (blue) in control and obstructed (10 days) bladders. Panel **F** shows the percentage of MeCP2 positive nuclei in bladders from sham-operated and obstructed (10 days) rats (n = 6 and 7, respectively).

Translational repression is an important mechanism of action of miRNAs and protein levels often change more than mRNA levels. We blotted for five of the targets surveyed above and for Ache because of its biological significance in bladder activation (Fig. 5A). Ache (Fig. 5A and summarized data in 5C), MeCP2 (Fig. 5A and summarized data in 5B), and Pnkd (Fig. 5A) were rapidly and forcefully repressed in obstructed bladders and the magnitude of change for MeCP2 and Pnkd exceeded the change seen at the mRNA level. Pten (Fig. 5A and 5D), Ripk2 and Sh3bp5 were only modestly reduced and Sh3bp5 reduction was not even significant (*p*>0.05 at 10 days). These findings supported a cause and effect relationship between miR-132/212 induction and repression of MeCP2 and Pnkd and possibly also of Ache.

To further substantiate the reduction of MeCP2, a nuclear protein that binds to methylated DNA and stimulates or represses transcription, we stained for DNA (blue) and MeCP2 (green) in sham-operated and obstructed bladders (Fig. 5E). Well over 50% of all nuclei of detrusor smooth muscle cells were positive for MeCP2 and the percentage of positive nuclei declined in obstructed bladders (Fig. 5F).

### Reduction of Ache correlates with synapse density in outlet obstruction and Ache is not induced in Dicer KO bladders

**Figure 6:**
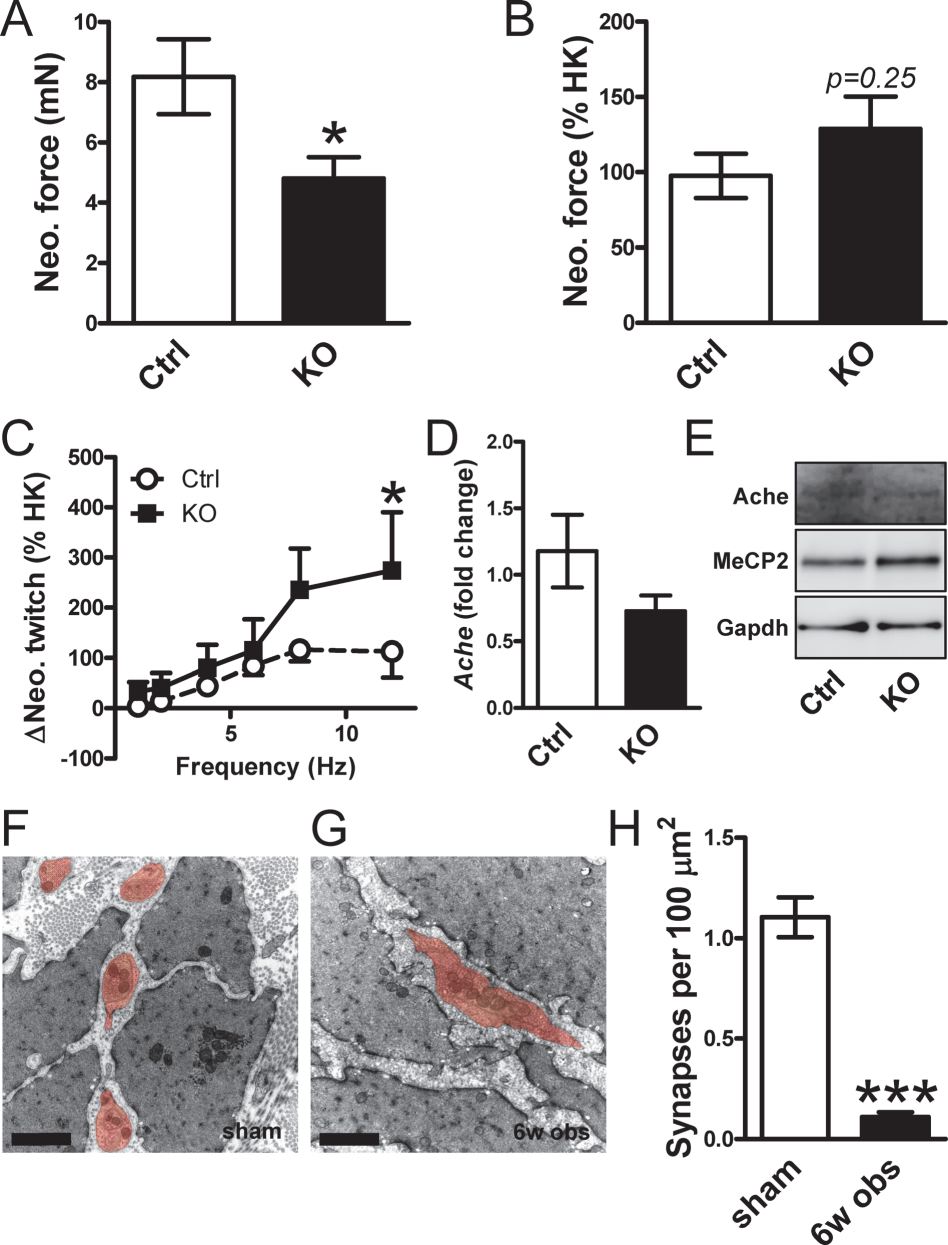
Smooth muscle-specific deletion of Dicer does not increase neostigmine-induced contraction or expression of Ache in the bladder. Panels **A** and **B** show contraction induced by the addition of the acetylcholine-esterase (Ache) inhibitor neostigmine in control and in Dicer knockout (KO) bladder strips. Force is given in mN (**A**) and relative to depolarization-induced contraction (**B**), respectively. Panel **C** shows the potentiation by neostigmine of twitches in response to electrical field stimulation (ΔNeo. twitch) in control and Dicer KO detrusor strips. Panels **D** and **E** show the mRNA and protein levels of Ache in control and Dicer KO bladders. Panels **F** and **G** show representative electron micrographs from sham-operated and obstructed (6 weeks) bladders. Neural varicosities are highlighted in red. Scale bars represent 1 μm. Panel **H** shows quantitative evaluation of synapse density in control and obstructed bladders. N = 6.

We hypothesized that miR-132/212 may regulate Ache in the bladder and that loss of miR-132/212-dependent repression of Ache might underlie the reported [3] impairment of cholinergic neuroeffector transmission 10 weeks after deletion of Dicer in the bladder. We therefore designed a set of experiments to directly address if Ache was more active and its expression higher in Dicer KO bladders. We first predicted that pharmacological inhibition of Ache should give rise to larger contraction and to more pronounced potentiation of electrically induced twitches in Dicer KO compared to control bladders on account of de-repression of Ache. Addition of the acetylcholine esterase inhibitor neostigmine resulted in a small contraction in both control and Dicer KO bladders. Contrary to our prediction, neostigmine-induced contraction was smaller in KO bladders (Fig. 6A). Contractility in Dicer KO bladders has been reported to be reduced [3] which would mask a relative increase of the neostigmine-induced contraction. We therefore normalized the neostigmine-induced contraction to depolarization-induced force in the same preparation, but we again failed to see an increase (Fig. 6B).

Acetylcholine is released from neural varicosities in the urinary bladder on electrical field stimulation and this gives rise to transient muscle twitches. We measured these twitches in control conditions, after addition of neostigmine and following desensitization of purinergic receptors using α, β-methylene-ATP. No difference between KO and control bladders was seen at any frequency when we plotted the neostigmine-induced change in twitch amplitudes (data not shown). No difference was moreover seen at frequencies between 1 and 10 Hz when neostigmine-induced potentiation was normalized to depolarization-induced contraction (Fig. 6C). A borderline significant increase was seen at 12 Hz, but further evidence against our hypothesis was the finding that *Ache* mRNA and protein were unchanged (Fig. 6D and E). In all, these findings argued that relief of miR-132/212-dependent repression of Ache does not underlie the reported cholinergic neuro-effector transmission defect in Dicer KO bladders.

To address if obstruction-induced loss of Ache was instead explained by a reduced synapse density we counted synapses using electron microscopy (Fig. 6F and G). Synapses were reduced by 90% (Fig. 6H) to be compared with the 86% reduction of Ache protein at this time after obstruction (c.f. Fig. 5C).

### MiR-132/212 and cell viability

**Figure 7:**
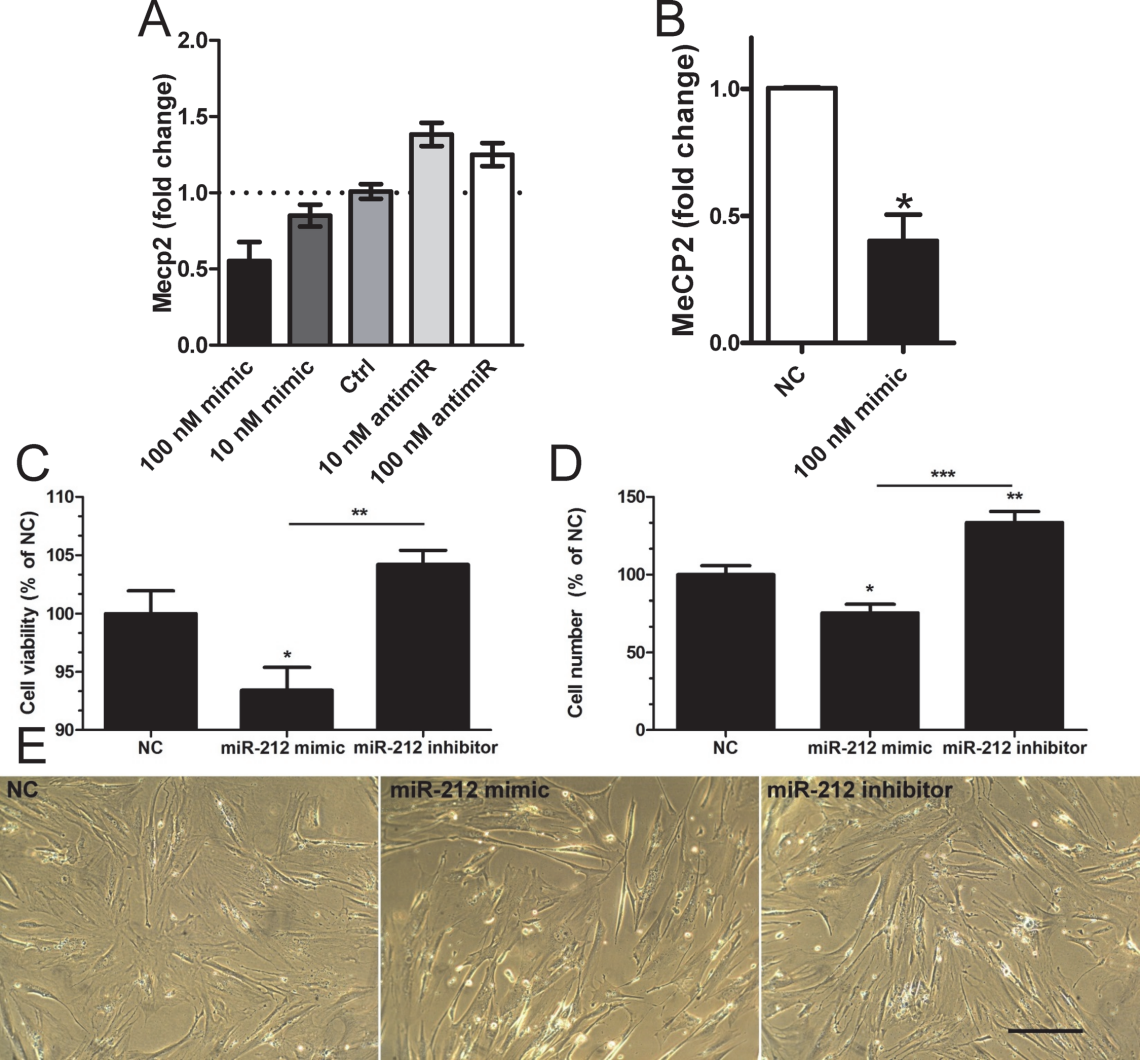
Transfection of miR-132/212 mimics and inhibitors affect MeCP2 expression and cell number in vitro. An initial dosing experiment performed in duplicate using human detrusor smooth muscle cells from one individual shows a reciprocal effect of miR-132 mimic and inhibitor on MeCP2 expression (**A**). The effect of 100 nM miR-132 mimic was confirmed using cells from three individuals (**B**). Panel **C** shows effect of miR-212 mimic and inhibitor (100 nM of each) on cell viability using the MTT assay. Panel **D** shows the effect of miR-212 mimic and inhibitor on cell number and panel **E** shows phase contrast light microscopic images of cells transfected with negative control (NC), miR-212 mimic and miR-212 inhibitor. The scale bar in **E** indicates 20 μm and n = 5 and 8 in **C** and **D**, respectively.

The lack of support for a role of miR-132/212 in regulation of Ache in the bladder led us to consider alternative roles. Using cultured human detrusor myocytes we transfected miR-132/212 mimics and antimirs. In a dosing experiment we found reciprocal changes in expression of the transcriptional regulator MeCP2 following transfection of miR-132 mimic and antimir (Fig. 7A). A similar trend was seen for miR-212 (not shown). The effect of 100 nM miR-132 mimic on MeCP2 expression was confirmed using cells from three different patients (Fig. 7B). We next examined effects on cell viability and cell number. MiR-212 mimic was found to reduce both viability and cell number (Fig. 7C and D), whereas the inhibitor increased cell number (Fig. 7D). These effects were apparent in bright field images of cells after transfection of miR-212 mimic and inhibitor compared to negative control (NC, Fig. 7E). Signs of apoptosis, such as cell membrane blebbing and cell shrinkage, were not apparent (Fig. 7E). MiR-132 inhibitor increased cell viability (+6.7±1.36%, *p*<0.01 vs. negative control), while miR-132 mimic was without effect (−0.7±1.6%, *p* = 0.75).

## Discussion

We document sizeable induction of miR-132/212 in rat bladder outlet obstruction. In fact, when expression levels on microrrays are compared, miR-132 is the most highly upregulated miRNA in this urological condition. Conversely, miR-29b and miR-29c, which play roles in matrix turnover, are the most highly repressed miRNAs [4]. We moreover demonstrate that induction of miR-132/212 is specific for the detrusor layer and does not occur in the mucosa. Smooth muscle cells represent the dominating cell type in the detrusor layer and 90% of all nuclei in obstructed bladders have a smooth muscle identity [25]. We also detect inducible expression of miR-132/212 in primary smooth muscle cells from the human urinary bladder. It is therefore reasonable to conclude that smooth muscle cells contribute to the increase in miR-132/212 in outlet obstruction even if basal expression in these cells may be low as suggested by our experiments on Dicer knockout mice. Contribution by some other cell type to miR-132/212 induction, including inflammatory cells, cannot be ruled out. Neurons are not likely to contribute because the cell bodies of detrusor neurons are located in the pelvic ganglia and we show that the number of synapses in the obstructed detrusor is reduced by 90%.

Creb binding to two CRE motifs upstream of miR-212 and one CRE motif upstream of miR-132 has been demonstrated by chromatin immunoprecipitation in rat neurons [6]. ACREB, which inhibits DNA binding of endogenous CREB, moreover blocked CREB-driven transcription of the miR-132 gene [6]. Experiments have also shown that the miR-132/212 cluster is induced by the dioxin receptor (Ahr) agonist TCDD in wild type but not in Ahr-deficient T-cells [18]. Together, these prior studies provide compelling evidence that the miR-132/212 cluster is regulated by Ahr/Creb. Our bioinformatics analysis of upstream mechanisms of miR-132/212 induction in outlet obstruction argues for involvement of Ahr. We detected increased nuclear staining for Ahr at 2 and 4 days of obstruction, and the dioxin receptor agonist TCDD was found to induce miR132/212 in cultured smooth muscle cells. The fold induction by TCDD was however modest compared to the large change observed in outlet obstruction, suggesting that some other mechanism may also contribute in the latter case. We failed to confirm activation of Creb1 by phosphorylation (S133), but an alternative Creb activation mechanism is possible. Forskolin, which activates Creb via cAMP accumulation, induced these miRNAs effectively *in vitro* as previously reported [6]. We failed to see an effect of the ER stressors brefeldin A and tunicamycin, which should activate ATF6. This leaves histone modifications or MeCP2/DNA methylation and Egr as possible contributors. Additional unknown influences [5] may also play a role. Our demonstration that serum stimulation, which induces growth of detrusor myocytes similar to outlet obstruction, increases miR-132/212, establishes a pattern between growth and miR-132/212 induction. Taken together, our analysis suggests involvement of Ahr and, possibly, Creb in miR-132/212 induction, but we cannot rule out additional critical influences.

The availability of a comprehensive survey of miRNA and mRNA expression in bladder outlet obstruction ([4], GEO: GSE47080) allowed us to correlate miR-132/212 levels with target mRNAs. We used the normalized sum of miR-132 and miR-212 because these miRNAs have the same seed sequences, but one may raise arguments against this approach. The expression of miR-132 in the bladder is higher than that of miR-212, making the contribution of miR-212 to the observed correlations modest. In view of the weaknesses with target prediction we focused our analyses on miR-132/212 targets that have been previously validated. *Mecp2, Ep300* and *Jarid1a* were chosen from work on the circadian clock [26] where conserved binding sites in the 3’UTR were demonstrated in each case. It was moreover shown, using 3’UTR luciferase assays, that miR-132 mimic reduced luciferase activity with wild type constructs, but not using constructs where the binding sites were mutated. Finally, using a tetracycline-inducible miR-132 transgenic mouse strain, the authors demonstrated that *Jarid1a, Mecp2* and *Ep300* are regulated by miR-132 *in vivo*. These targets therefore meet very rigorous validation criteria, in keeping with the strong correlations observed here. *Pnkd, Sh3bp5* and *Ripk2* were chosen from a list of high confidence targets identified by a RISC-trap approach [27], but 3’UTR luciferase assays have yet to be performed. *Foxo3, Pten* and *Rasa1*, finally, were all validated using reporter assays [14], [19], [13]. Thus, while the targets studied here have binding sites for miR-132/212 and, in the majority of cases, have been stringently validated, three of them meet only basic validation criteria. Overall, however, our findings at the mRNA level agree well with prior studies in other cells and tissues. The only possible exceptions are *Pten* and *Rasa1*, but this could be due to overriding influences in our model. We also note that the original study on miR-132/*Rasa1* only reported an effect on the Rasa1 protein [13]. While the correlations identified here by us further support prior miR-132/212 target studies, they do not firmly establish causality between miR-132/212 induction and target repression in bladder outlet obstruction. Experiments to firmly establish cause and effect relationships in outlet obstruction would, in our view, require use of miR-132/212 knockout mice.

The most compelling associations at the protein level in outlet obstruction were obtained for Ache, MeCP2 and Pnkd. Pnkd is referred to as Myofibrillogenesis Regulator-1 and this protein has been shown to regulate F- to G-actin ratios and cardiac hypertrophy. While Pnkd may be of importance in this regard also in smooth muscle, we did not follow up on this protein because of difficulties with detection in human detrusor myocytes. Ache was previously validated as a target of miR-132 using multiple approaches [12] and induction of miR-132/212 seemingly correlated with repression of Ache in our obstruction model. These findings raised the possibility that de-repression of Ache might underlie the reported [3] cholinergic neuro-effector transmission defect in smooth muscle-specific Dicer KO bladders. Several lines of evidence however argued against this possibility. We first found that contraction in response to neostigmine was reduced rather than increased in KO bladders where miR-132/212 were clearly, albeit modestly, reduced. We moreover found that neostigmine-mediated potentiation of cholinergic twitches was unchanged and that Ache mRNA and protein were largely unaffected. Dicer KO affects many miRNAs and the reductions of miR-132 and miR-212 were small, so we cannot rule out a modulating influence of miR-132/212 on Ache in the bladder. It is however unlikely that that miR-132 and miR-212 are responsible for the neuro-effector transmission defect that we have previously reported in Dicer KO bladders [3]. The reduction of Ache in outlet obstruction moreover correlates with a reduction of synapse density. Taken together these findings argue against a role of Ache in the cholinergic neurotransmission defect in miRNA depleted mouse bladders [3] and rather validates use of Ache for quantitation of bladder innervation in outlet obstruction.

MeCP2 is a methyl-CPG-binding protein that regulates transcription [28]. Recent work has demonstrated that MeCP2 is involved in miRNA processing via an interaction with DGCR8 [29]. The function of MeCP2 in smooth muscle has not been addressed but this gene is mutated in Rett syndrome, a neurodevelopmental disorder with widespread autonomic dysfunction. Prior studies have demonstrated cell autonomous roles of MeCP2 in both skeletal and cardiac muscle [30]. MeCP2 positively regulates smooth muscle α-actin (*Acta2*) expression in fibroblasts [31] via binding to the gene (*Acta2*) promoter. Repression of MeCP2 is therefore likely to have repercussions for detrusor function. If and how MeCP2 affects smooth muscle function and development therefore needs to be directly addressed.

Prior work has shown that miR-132 and miR-212 regulate cardiomyocyte hypertrophy by targeting FoxO3 and calcineurin-A/NFAT signaling [14]. Since bladder outlet obstruction is associated with considerable hypertrophy of detrusor myocytes [32] it is conceivable that miR-132/212 may play a similar role in the obstructed bladder. In support of this possibility we found a significant correlation between miR-132/212 and FoxO3, consistent with the findings by Ucar *et al.* [14]. However, our transcription factor binding site analysis did not support enrichment of NFAT binding sites in differentially expressed genes at 10 days of obstruction (*p* = 0.98). This speaks against a critical role of NFAT in gene expression changes seen at this time. Others have demonstrated modest NFAT activation following partial bladder outlet obstruction [33], further arguing that it may be worthwhile to examine the effect of miR-132/212 knockout and overexpression on the response of the urinary bladder to outlet obstruction. Here we demonstrate that transfection of miR-212 mimic and inhibitor has opposing effects on detrusor cell number *in vitro*. This is concordant with the results of Choe et al. [21] on miR-132 mimic and antimir transfection in vascular smooth muscle cells. MiR-132/212 induction may therefore limit the hyperplasia that occurs following outlet obstruction and, possibly, simultaneously promote hypertrophy. It is important to note, however, that the effects of miR-132/212 mimics and blockers on detrusor cell number may be mediated by miRNA targets other than those studied here.

In conclusion, our work has demonstrated induction of miR-132/212 in bladder outlet obstruction. This correlates with changes in target protein expression in the bladder and may limit detrusor cell number. Induction of miR-132/212 in outlet obstruction is probably not unique for rats because preliminary miRNA profiling indicates similar changes in hypertrophic bladders from humans [34]. Here, we demonstrate induction of these miRNAs in growth-stimulated human detrusor cells and find that previously validated targets of miR-132/212 change in the predicted manner on miR-132/212 induction. The present study therefore sketches a signaling pathway elicited by obstruction that involves Ahr/Creb-mediated induction of miR-132/212, miR-132/212-dependent repression of MeCP2, Ep300 and Jarid1a and reduced cell proliferation. The causality between these events however needs to be further corroborated.
